# Beware of bile peritonitis caused by kinking of the self-expandable metal stent during endoscopic ultrasound-guided hepaticogastrostomy

**DOI:** 10.1055/a-2689-3676

**Published:** 2025-09-04

**Authors:** Masaki Miyazawa, Hidenori Kido, Kazuki Nagai, Tomoyuki Hayashi, Shinya Yamada, Hajime Takatori, Taro Yamashita

**Affiliations:** 1 88335 Gastroenterology, Kanazawa University Hospital, Kanazawa, Japan

Self-expandable metal stents (SEMSs) are often used for endoscopic ultrasound-guided hepaticogastrostomy (EUS-HGS), but bile peritonitis due to stent dislocation or cover breakage can be a serious adverse event 1 2 3 . While kinking of the SEMS is occasionally a problem with transpapillary stenting, there have been no reports of kinking of the SEMS in EUS-HGS. Here, we report a case in which a SEMS used in EUS-HGS kinked and caused bile peritonitis ( Video 1 ).

Following successful endoscopic ultrasound-guided hepaticogastrostomy using a self-expandable metal stent (SEMS) for biliary obstruction due to hepatocellular carcinoma, bile peritonitis occurred, which was found to have been caused by kinking of the SEMS.Video 1

A 50-year-old man on drug treatment for hepatocellular carcinoma that was occupying a large portion of his liver developed obstructive jaundice. The left intrahepatic bile duct was dilated and the left hepatic duct was obstructed by the tumor ( Fig. 1 ). Because of the long stenosis and the highly compressed duodenum owing to the tumor, transpapillary stenting was considered difficult, so we performed EUS-HGS. The B3 intrahepatic bile duct was punctured with a 19-gauge needle ( Fig. 2 **a** ), and a guidewire was inserted. After the fistula had been dilated ( Fig. 2 **b** ), a SEMS (8 × 100 mm; HANAROSTENT Biliary Partial Cover Benefit; Boston Scientific, Massachusetts, USA) was placed ( Fig. 2 **c** ). All steps in the procedure were completed without any difficulty; however, 5 days after the creation of the EUS-HGS, the patient complained of fever and abdominal pain, and computed tomography showed fluid retention between the liver and stomach ( Fig. 3 **a** ). Furthermore, kinking of the SEMS at the bile duct puncture site was observed ( Fig. 3 **b** ), and it was thought that bile peritonitis had developed owing to poor bile drainage caused by the kinking. Reintervention was attempted through the fistula, but the catheter could not be passed through the kinked site of the SEMS ( Fig. 3 **c** ), so percutaneous bile leak drainage and percutaneous transhepatic biliary drainage were performed.

**Fig. 1:**
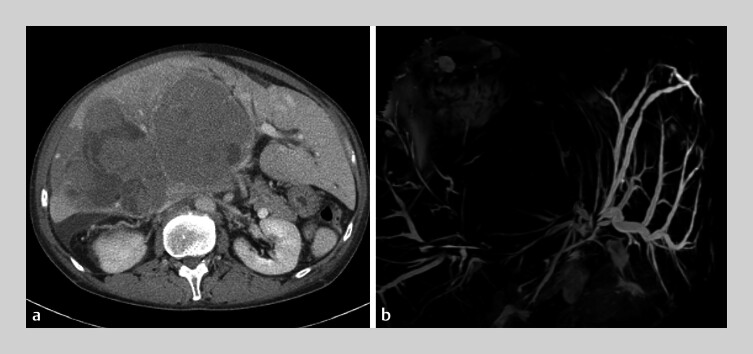
Imaging findings in a patient with hepatocellular carcinoma occupying a large portion of the liver showing the dilated left intrahepatic bile duct on: **a** computed tomography; **b** magnetic resonance cholangiopancreatography.

**Fig. 2:**
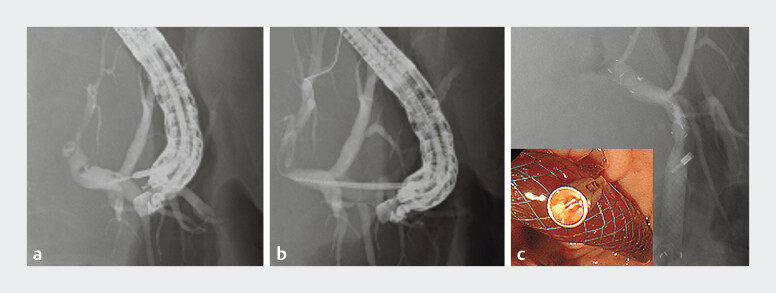
Fluoroscopic images during endoscopic ultrasound-guided hepaticogastrostomy showing: **a** intrahepatic bile duct puncture being performed with a 19-gauge needle; **b** dilation of the fistula with a drill dilator; **c** placement of a self-expandable metal stent from the intrahepatic bile duct to the stomach.

**Fig. 3:**
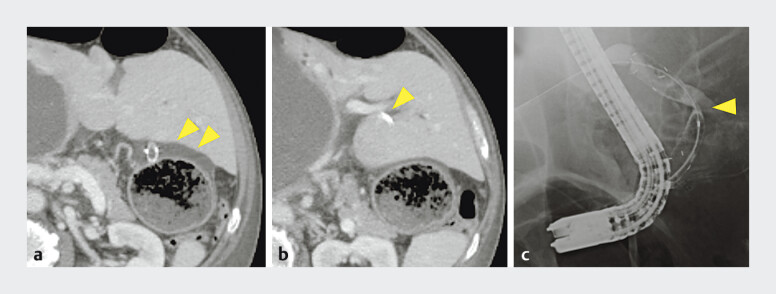
Images after the patient had developed bile peritonitis showing: **a** on computed tomography 5 days after the creation of the endoscopic ultrasound-guided hepaticogastrostomy, fluid retention between the liver and stomach (arrowhead); **b** kinking of the self-expandable metal stent (SEMS) at the bile duct puncture site (arrowhead); **c** on fluoroscopic image, reintervention being attempted through the fistula, but with the catheter unable to pass through the kinked site of the SEMS (arrowhead).

Even with the procedural success of SEMS placement in EUS-HGS, bile peritonitis due to kinking of the SEMS should be kept in mind.

Endoscopy_UCTN_Code_TTT_1AR_2AD

## Notes

Endoscopy E-Videos https://eref.thieme.de/e-videos
*E-Videos* is an open access online section of the journal *Endoscopy* , reporting on interesting cases and new techniques in gastroenterological endoscopy. All papers include a high-quality video and are published with a Creative Commons CC-BY license. Endoscopy E-Videos qualify for HINARI discounts and waivers and eligibility is automatically checked during the submission process. We grant 100% waivers to articles whose corresponding authors are based in Group A countries and 50% waivers to those who are based in Group B countries as classified by Research4Life (see: https://www.research4life.org/access/eligibility/ ). This section has its own submission website at https://mc.manuscriptcentral.com/e-videos .
